# Gender disparity between first and senior authors on liver cancer research in the top journals of Gastroenterology and Hepatology

**DOI:** 10.1371/journal.pone.0295648

**Published:** 2024-05-31

**Authors:** Huiqin Shi, Huan Xu, Shu Huang, Zhenju Tan, Xinyue Ma, Han Zhang, Wei Zhang, Lei Shi, Xiaolin Zhong, Muhan Lü, Xia Chen, Xiaowei Tang

**Affiliations:** 1 Department of Gastroenterology, The Affiliated Hospital of Southwest Medical University, Region Jiangyang, Luzhou, 646099, Sichuan Province, China; 2 Department of Gastroenterology, The People’s Hospital of Lianshui, Lianshui, 223499, Huaian, Jiangsu Province, China; 3 Department of Gastroenterology, Clinical Medical College and The First Affiliated Hospital of Chengdu Medical College, Xindu District, Chengdu City, 610500, Sichuan Province, China; Universal Scientific Education and Research Network, UNITED KINGDOM

## Abstract

**Background:**

Gender disparity is pervasive in academic medicine. This study aimed to assess the disparity between men and women with regard to first and senior author positions in primary studies on liver cancer over the last two decades.

**Methods:**

We conducted a review of articles published in high-impact factor journals of the field of Gastroenterology and Hepatology in 2005, 2010, 2015 and 2020. First and senior authors of all ages were considered as the study population. The authors’ genders were determined using the online artificial intelligence tool genderize.io (https://genderize.io/). The disparity between men and women authors was assessed using the linear-by-linear association test.

**Results:**

665 original articles from 10 journals were reviewed. The point prevalence of first women authors was 25.0% compared with 75.0% for men. The point prevalence of senior women authors was 16.3% compared with 83.7% for men. From 2000 to 2020, the proportion of first women authors increased 14.4% to 26.8% compared with 85.6%-73.2% for men (P = 0.009), and the proportion of senior women authors increased from 7.4% to 19.5%, compared with 92.6%-80.5% for men (P = 0.035). The factor independently associated with a reduced representation of women among first authors was the region of author. The factor independently associated with a reduced representation of women among senior authors was the impact factor of journals.

**Conclusion:**

The findings indicated a remarkable increase in the proportion of women, both first and senior authors, over the past two decades in the field of liver cancers. However, the representation of women authors in this area is far less than that of men.

## Introduction

Gender diversity can drive innovation, improve collaboration, and advance health care. Although women have made substantial progress in academic medicine, they are still underrepresented in most fields [1]. Women are active and prolific contributors to the field of Gastroenterology and Hepatology [2]. However, statistics showed that only 19% of practicing gastroenterologists in 2019 were women in the United States (US) [3]. In addition, only 14.5% of women senior authors in the US had published original articles in major academic gastroenterology journals [4]. These studies indicated that women also face gender inequity in the field of Gastroenterology and Hepatology.

Generally speaking, high-impact and original research publications are regarded as the key to academic advancement and are related to promotion and research funding [5]. Recent studies indicated that women were significantly less likely than men to publish in quality gastroenterology journals [6]. If effective measures are not taken, it will be challenging to improve gender inequity. Liver cancer is an important research direction in the field of academic medicine. It is understood that there are few studies focusing on gender disparity in this area.

Therefore, we sought to investigate the disparity between men and women regarding first and senior author positions in articles on liver cancer published in top-quality journals of Gastroenterology and Hepatology over the past 20 years.

## Methods

### Study design, study period and data sources

We performed a longitudinal analysis of the prevalence of men and women first and senior authors of primary studies on liver cancer over the last two decades. Although this longitudinal study is neither a classic epidemiological study, nor a bibliometric study, nor a scientometric study, we used the cohort study design as main model for conducting and reporting this study. Accordingly, our report is based on STROBE standards for cohort studies. Data on authors were retrieved from a review of articles published in top-quality journals of the field of Gastroenterology and Hepatology in 2000, 2005, 2010, 2015 and 2020. Those journals included: *the Journal of Hepatology (J Hepatol)*, *Gut*, *Gastroenterology*, *American Journal of Gastroenterology (AJG)*, *Alimentary Pharmacology & Therapeutics (APT)*, *Journal of Gastroenterology (J Gastroenterol)*, *Hepatology*, *Journal of Hepato-Biliary-Pancreatic Sciences (JHBPS)*, *Liver International (Liver Int)*, *and Liver Transplantation (Liver Transpl)*. The classification of those journals as top journals was based on the Clarivate 2020 Journal Citation Reports [7].

### Study population

Inclusion and exclusion criteria: The type of articles included was original researches only, as it best representative of the real academic research level of the researchers, and other types of articles such as meta-analyses, case reports, systematic reviews and editorials were excluded. And these journals that published original researches on liver cancer must be top-journals in the field of Gastroenterology and Hepatology, and have been in circulation for at least 20 years. As the first author is a junior academic who wrote the article and the last author is a senior academic who may have conceived and funded the research, these two positions are generally regarded as the most important [8]. Original articles that did not provide the names of the first or senior authors, as well as articles with only one author, were initially excluded.

Study population: First and senior authors of all ages were considered as the study population. Women first and senior authors were the primary study population, while the men first and senior authors were a comparative population.

### Data collection

Through the official website of the journals, we collected all original articles published in the 10 journals that met the above criteria between January and December in 2000, 2005, 2010, 2015 and 2020, respectively, and recorded the following information for each original article: journal name, title of the original articles, abstract, time of publication, first and last names of first and senior authors, country and affiliations of senior authors (using correspondence address of the corresponding author to determination) and the impact factor (IF) of the journals. Then, we used "liver cancer", "hepatocellular carcinoma", “hepatic carcinoma” or “hepatoma” as keywords for article title or abstract to screen out articles related to liver cancer among all original articles in our collection.

The genderize.io database (https://genderize.io/) was used to identify the gender of first and senior authors based on their first names. We set a 90% probability threshold to determine the gender of the authors and only included articles where the probability of the author’s gender was greater than or equal to 90%. When the aforementioned tools failed to identify gender of the author, we visited the institute’s website or used a search engine to find the author’s full name, which might provide some information indicating the gender [2]. If the gender could not be determined using above-mentioned methods, the author was excluded.

### Outcome

The main outcomes were the change in the representation of women first authors and women senior authors over time compared to men authors of the same position. Secondary outcomes were factors associated with gender disparity regarding the changes.

### Statistical analysis

All data were stored in Excel. All analyses were performed by SPSS Statistics (version 26.0) and graphics were drawn by GraphPad Prism 8. We calculated the percentage point difference between first and senior authors. A linear-by-linear association test was used to analyze the trend of woman proportion over time, while logistic regression analysis was performed to determine the factors impacting gender differences between first and senior authors. For the first author, candidate factors were nationality of first authors, journal IFs and gender of senior authors, and the result was gender of first authors. For senior authors, candidate factors were journal IFs, nationality of senior authors, and gender of first authors, with the result being gender of senior authors. Odds ratios (OR) and 95% confidence intervals (CI) were used to report these effect magnitude estimates. When P <0.05, OR>1 indicated that the factor was conducive to the improvement of the proportion of women authors, and OR<1 indicated that the factor was not conducive to the increase of the proportion of women authors.

### Ethical issues

Given that this study is based on the authors’ names provided in published articles, ethical approval is not required for this study.

## Results

### Study selection and characteristics of the study population

**Fig 1:**
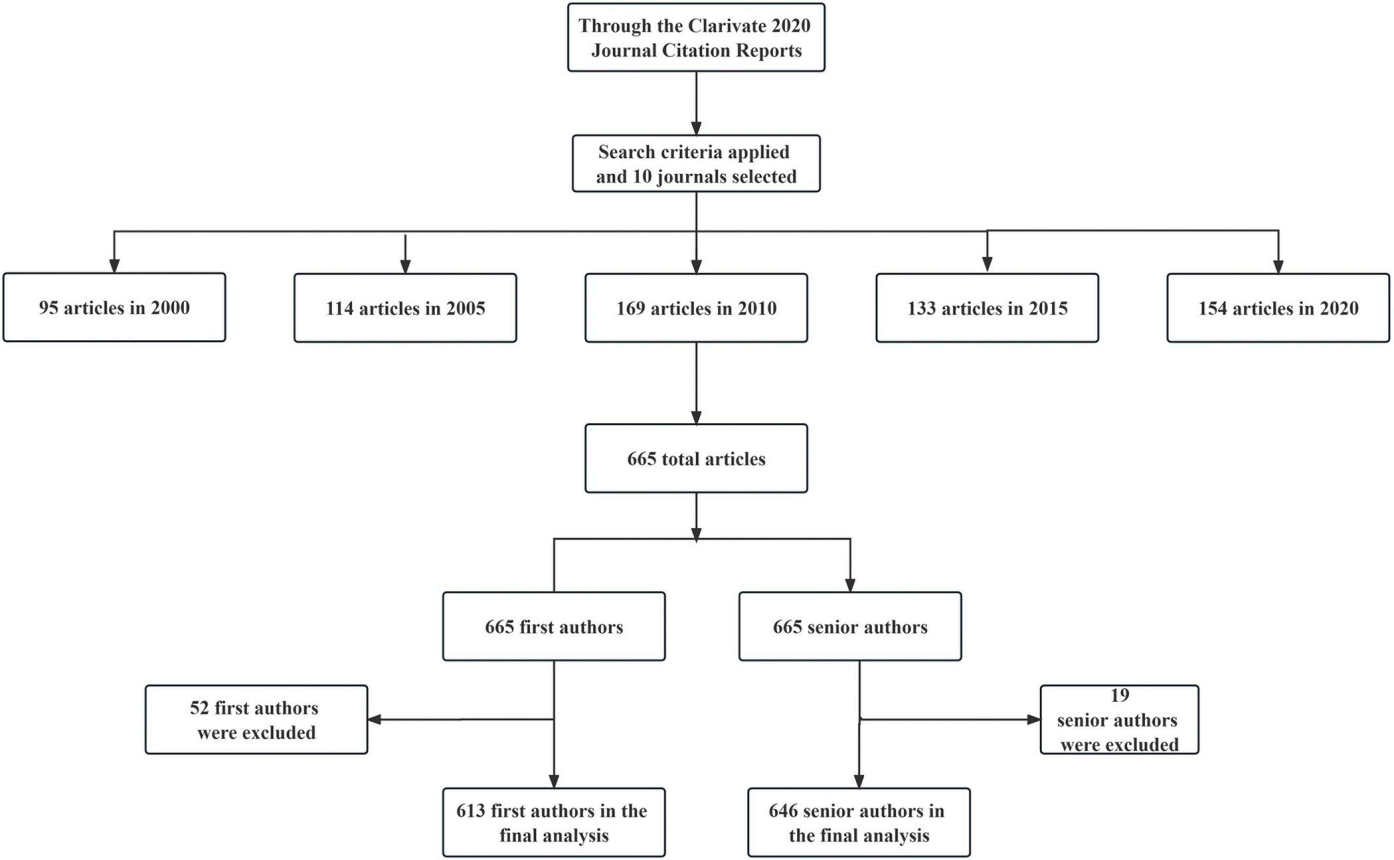
Flow chart on the selection process for articles from where study participants were retrieved. 10 journals were *the Journal of Hepatology*, *Gut*, *Gastroenterology*, *American Journal of Gastroenterology*, *Alimentary Pharmacology & Therapeutics*, *Journal of Gastroenterology*, *Hepatology*, *Journal of Hepato-Biliary-Pancreatic Sciences*, *Liver International*, *and Liver Transplantation*.

We have included 1330 authors from 665 original research articles published in 10 top-quality journals of the field of Gastroeneterology and Hepatology between 2000 and 2020. 52 (7.8%) first authors and 19 (2.9%) senior authors were excluded because their gender could not be identified. Of the 1,259 gender-specific authors, 613 were first authors and 646 were senior authors (Fig 1).

### Gender distribution of first and senior author positions over time

There were 258 (20.5%) women authors and 1001 (79.5%) men authors. Based on the overall publication time (five-year intervals), our study showed the number and percentage of women authors of original articles in each journal who served as the first/senior author. Proportions of women first and senior authors were highest in *APT* (n = 7/17, 41.2%) and *Gastroenterology* (n = 10/40, 25.0%), respectively (S1 Table). From 2000 to 2020, the overall rate of first authors ranged from 14.4% to 26.8% for women, and from 85.6% to 73.2% for men. Interestingly, the proportion of women first authors increased rapidly between 2010 and 2015, from 22.6% to 32.5%, while the proportion of men first authors decreased from 77.7% to 67.7% (P = 0.009). Similarly, from 2000 to 2020, the overall rate of senior authors ranged from 7.4% to 19.5% for women, and from 92.6% to 80.5% for men (P = 0.035). Despite this, the proportion of women authors who published original articles on liver cancer was still much lower than that of men authors, either as first or senior authors (P = 0.001, Table 1).

**Table 1:** Percentage of women first and senior authors on liver cancer publications between 2000 and 2020.

	First authors		Senior authors		Total	
Year	Woman	Man	Woman	Man	Woman	Man
2000	13 (14.4)	77 (85.6)	7 (7.4)	87 (92.6)	20 (10.9)	164 (89.1)
2005	25 (22.7)	85 (77.3)	18 (15.9)	95 (84.1)	43 (19.3)	180 (80.7)
2010	26(22.6)	89 (77.4)	24(18.8)	104 (81.2)	50 (20.6)	193 (79.4)
2015	52(32.5)	108 (67.5)	27(16.7)	135 (83.3)	79 (24.5)	243 (75.5)
2020	37(26.8)	101 (73.2)	29(19.5)	120 (80.5)	66 (23.0)	221 (77.0)
Total	153 (25.0)	460 (75.0)	105(16.3)	541 (83.7)	258 (20.5)	1001 (79.5)
P-value	**0.009**		**0.035**		**0.001**	

**Fig 2:**
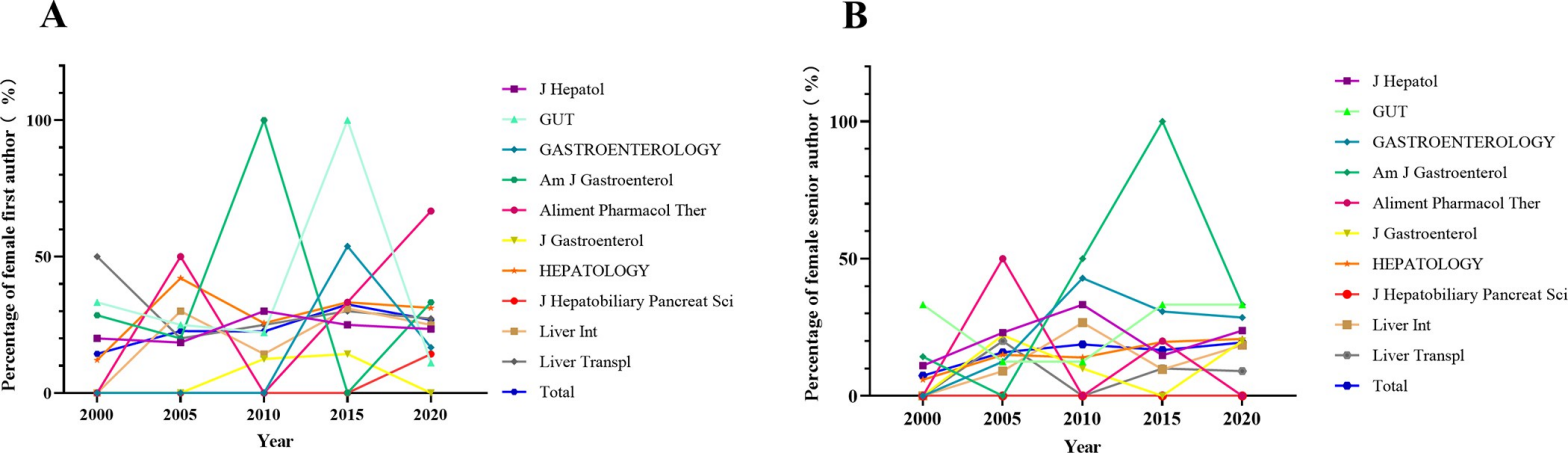
(A) Trends in the proportion of women first authors over time (both each journal and total); (B) Trends in the proportion of women senior authors over time (both each journal and total).

Although the proportion of women authors in most journals has risen over time, *Gut and Liver Transpl* saw a decrease in the proportion of women first authors (*Gut*, from 33.3% to 11.1%; *Liver Transpl*, from 50.0% to 27.3%), while the proportion of women senior authors in *Gut* did not change significantly from 2000 to 2020 (from 33.3% to 33.3%). We collected only one original article on liver cancer written by a woman first author in the *JHBPS* journal. This may indicate that few women researchers publish original articles on liver cancer in *JHBPS* as first or senior authors. Overall, the percentage of women authors increased significantly over time, and the difference was statistically significant (Fig 2).

### Factors associated with a lower representation of women than men

The results of the logistic regression analysis suggested that it was more difficult for women from Asia to publish as first authors in high-impact journals compared with their counterparts in North America (OR = 0.33; 95% CI, 0.706–1.855; P = 0.001). Among first authors in Europe and North America, there was no statistical difference in the proportion of women who published original articles (OR = 1.14; 95% CI, 0.706–1.855; P = 0.584). The gender of first author was not associated with the gender of senior author (OR = 1.04; 95% CI, 0.623–1.734; P = 0.882) and the IF of journals (OR = 0.98; 95% CI, 0.826–1.152; P = 0.773). For senior author, there was no correlation between the publication of articles by women authors and the author’s region (OR = 1.39; 95% CI, 0.759–2.543; P = 0.287; OR = 0.71; 95% CI, 0.383–1.300; P = 0.264) or the gender of first author (OR = 1.05; 95% CI, 0.624–1.749; P = 0.868). Women senior authors were more likely to publish original articles in journals with higher-IFs compared with men authors (OR = 1.27; 95% CI, 1.064–1.525; P = 0.008) (Table 2).

**Table 2:** Logistic regression analysis of factors associated with the gender of women first and senior authors.

Variable	First author		Senior author	
	P-value	OR (95% CI)	P-value	OR (95% CI)
Journal impact factor	0.773	0.98 (0.826–1,152)	**0.008**	1.27 (1.064–1.525)
Region of author				
North America	Reference		Reference	
Europe	0.584	1.14 (0.706–1.855)	0.362	1.30 (0.740–2.280)
Asia	**0.001**	0.33 (0.206–0.516)	0.183	0.69 (0.396–1.193)
First author				
man			Reference	
woman			0.868	1.05 (0.624–1.749)
Senior author				
man	Reference			
woman	0.882	1.04 (0.623–1.734)		

## Discussion

### Principal findings

In this study of gender disparity in the original articles of liver cancer in the past 20 years, we confirmed that the proportion of women first (14.4% to 26.8%) and senior authors (7.4% to 19.5%) publishing original research articles increased significantly over time, but remained well below that of men authors in the same position (P = 0.001). The difference was that *Gut* saw a decrease in the proportion of women first authors, from 33.3% to 11.1% as well as *Liver Transpl*, from 50.0% to 27.3%. For women senior authors, they were more likely to publish in higher-IF journals (P = 0.008). While for women first authors, it was more challenging for them from Asia to publish original articles on liver cancer (P = 0.001).

### Reasons for the gender inequity

Our research showed that the status of women author in the field of liver cancer has improved, but gender inequity has not been completely eliminated in academic medicine. A previous oncology study indicated a marked increase in the number of women authors from 25.5% in 2002 to 31.7% in 2018. [9] Similarly, Mehran et al. reported that the proportion of women first authors in cardiology increased from 22% in 2011 to 35% in 2020 [10]. A recent article on women authors in infectious diseases journals also confirmed that while the proportion of women authors has increased, it was still lower than that of men authors [11]. In addition, women are underrepresented in the field of Gastroenterology and Hepatology, especially women senior authors [2,12]. Our findings are consistent with those of previous studies.

There was a significant growth in woman medical practitioners in the field of gastroenterology at the beginning of the 20th century [13], but women researchers with academic productivity in the corresponding field grew at a slow pace. The leading known reason is gender bias. Studies have shown that double-blinded reviews have contributed to the growth of women first author papers. When the author’s gender is known, research submitted by men authors is considered to be of a higher quality [14,15]. When the percentage of women editors increased, so did the percentage of women first authors and women senior authors [11]. However, women editors were still underrepresented [16], which to some extent made it more difficult for women authors to publish original research. The proportion of women first authors in *Liver Transpl* has declined from 50% to 30% over the past 20 years, possibly due to the fact that women surgeons were less likely to receive research grants than their men counterparts [17]. A previous study in the Hepatology Medical field confirmed this, with 55.7% of men first authors received government funding for their research compared with only 38.5% of women authors. Government funding was more likely to be provided to original research when authors were men rather than women [2]. Compared with men, women pay more attention to the workplace climate [18]. Women are often seen as underachievers who undervalue their own abilities, which lead them to leave academia [19].

Many career challenges faced by women, such as excessive familial responsibilities, make it difficult for them to focus on academic research [20]. Studies have found that women are more likely to spend more time every week on family activities and raising children than men, and most mothers believe that having children substantially slows their career progress [21,22]. In addition, young women continue to experience difficulty in balancing their work and domestic responsibilities whereas young men doctors focus more on factors directly related to work. A critical balance between work and family conflicts may cause women to drop out of academic research [23,24].

The underrepresentation of women senior authors has also been regarded as a hindrance to the academic progress of women. On the one hand, women publish fewer articles than men [25], and women authors as a group are less productive than their men counterparts [26]. Consequently, women may take longer than men to attain leadership positions. Since most women enter the field of gastroenterology and hepatology later, junior women authors have not yet transitioned to senior authors [4]. Lack of guidance from women senior authors also negatively affects the proportion of women first authors [6]. Interestingly, women as senior authors were slightly more likely to be published in high-IF journals than men in our study (OR = 1.27; 95% CI, 1.064–1.525; P = 0.008). One possible reason may be that women have stricter performance standards. For instance, the sample size of clinical research published by women was larger than that published by men [6].

Furthermore, our study showed that the lowest percentage of women author was from Asia and the highest was from Europe, compared to North America. This could be related to the level of regional development. One research suggested that women from low and middle-income countries were at a disadvantage in the impact factors of journals they published [27]. Nevertheless, women senior authors have accumulated enough international influence, expertise and research experience over their careers to be less adversely affected by regional differences [28].

### Strengths and limitations

As one of the important diseases in the field of Gastroenterology and Hepatology, liver cancer is an important research direction in the field of academic medicine. However, few studies reported gender disparity in this field. Our study confirmed for the first time that women authors are underrepresented in this field. At the same time, it is the first study to investigate authors’ gender inequity in top-quality journals of the field of Gastroenterology and Hepatology, which is more representative than previous studies and draws attention to the academic plight of women authors in this field.

There are several limitations to our study. The possibility of misidentifying an author’s gender is difficult to eliminate. And the gender identification tool we used was more suitable for Western authors while authors from other regions could only be clarified in other ways, such as the institute’s website and search engines. In addition, the genderize.io database which does gender confirmation based on the author’s name, does not distinguish cisgender individuals from transgender and intersex people.

## Conclusion

Overall, our study mainly suggested that there was a gender disparity on liver cancer in top-quality journals of Gastroenterology and Hepatology from 2000 to 2020. Although the underrepresentation of women has been improved, it has not been eliminated in this field. Greater attention should be devoted to this area to clarify the causes of gender disparity and find solutions to close the woman-man gap.

## Supporting information

S1 Table Distribution of gender among first authors and senior authors on top 10 journals about liver cancer in the field of Gastroeneterology and Hepatology. (DOCX)

S1 Data The original data presented in this study. (XLSX)
