# Supplementary material for: Gender disparity between first and senior authors on liver cancer research in the top journals of Gastroenterology and Hepatology
Source: PLoS One. 2024 May 31;19(5):e0295648. doi: 10.1371/journal.pone.0295648 (PMC11142674; doi:10.1371/journal.pone.0295648)
Supplement: S1 Table — (DOCX) [file pone.0295648.s001.docx]

| Journal | Female, n (%) | Year | | | | | Total |
| --- | --- | --- | --- | --- | --- | --- | --- |
|  |  | 2000 | 2005 | 2010 | 2015 | 2020 |  |
| JOURNAL OF HEPATOLOGY | First author | 5 (20.0) | 5 (18.5) | 6 (30.0) | 7 (25.0) | 4 (23.5) | 27 (23.1) |
|  | Senior author | 3 (11.1) | 6 (23.1) | 7 (33.3) | 4 (14.8) | 5 (23.8) | 25 (20.3) |
| GUT | First author | 1 (33.3) | 2 (25.0) | 2 (22.2) | 3 (100.0) | 1 (11.1) | 9 (28.1) |
|  | Senior author | 1 (33.3) | 1 (12.5) | 1 (12.5) | 1 (33.3) | 3 (33.3) | 7 (22.6) |
| GASTROENTEROLOGY | First author | 0 (0.0) | 0 (0.0) | 0 (0.0) | 7 (53.8) | 1 (16.7) | 8 (21.6) |
|  | Senior author | 0 (0.0) | 1 (12.5) | 3 (42.9) | 4 (30.8) | 2 (28.6) | 10 (25.0) |
| AMERICAN JOURNAL OF GASTROENTEROLOGY | First author | 2 (28.5) | 1 (20.0) | 2 (100.0) | 0 (0.0) | 2 (33.3) | 7 (33.3) |
|  | Senior author | 1 (14.3) | 0 (0.0) | 1 (50.0) | 1 (100.0) | 2 (33.3) | 5 (23.8) |
| ALIMENTARY PHARMACOLOGY ＆ THERAPEUTICS | First author | 0 (0.0) | 1 (50.0) | 0 (0.0) | 2 (33.3) | 4 (66.7) | 7 (41.2) |
|  | Senior author | 0 (0.0) | 1 (50.0) | 0 (0.0) | 1 (20.0) | 0 (0.0) | 2 (11.8) |
| JOURNAL OF GASTROENTEROLOGY | First author | 0 (0.0) | 0 (0.0) | 1 (12.5) | 1 (14.3) | 0 (0.0) | 2 (5.6) |
|  | Senior author | 0 (0.0) | 2 (22.2) | 1 (10.0) | 0 (0.0) | 1 (20.0) | 4 (10.0) |
| HEPATOLOGY | First author | 4 (12.1) | 8 (42.1) | 11 (25.6) | 20 (33.3) | 15 (31.2) | 58 (28.6) |
|  | Senior author | 2 (5.9) | 3 (15.0) | 7 (14.0) | 12 (19.7) | 10 (20.8) | 34 (16.0) |
| JOURNAL OF HEPATO-  BILIARY-PANCREATIC SCIENCES | First author | 0 (0.0) | 0 (0.0) | 0 (0.0) | 0 (0.0) | 1 (14.3) | 1 (5.9) |
|  | Senior author | 0 (0.0) | 0 (0.0) | 0 (0.0) | 0 (0.0) | 0 (0.0) | 0 (0.0) |
| LIVER INTERNATIONAL | First author | 0 (0.0) | 6 (30.0) | 2 (14.3) | 9 (31.0) | 6 (25.0) | 23 (25.0) |
|  | Senior author | 0 (0.0) | 2 (9.1) | 4 (26.7) | 3 (9.7) | 5 (18.5) | 14 (14.0) |
| LIVER TRANSPLANTATION | First author | 1 (50.0) | 2 (20.0) | 2 (25.0) | 3 (30.0) | 3 (27.3) | 11 (26.8) |
|  | Senior author | 0 (0.0) | 2 (20.0) | 0 (0.0) | 1 (10.0) | 1 (9.1) | 4 (9.8) |
| TOTAL | First author | 13 (14.4) | 25 (22.7) | 26 (22.6) | 52 (32.5) | 37 (26.8) | 153 (25.0) |
|  | Senior author | 7 (7.4) | 18 (15.9) | 24 (18.8) | 27 (16.7) | 29 (19.5) | 105 (16.3) |
|  | Authors | 20 (10.9) | 43 (19.3) | 50 (20.6) | 79 (24.5) | 66 (23.0) | 258 (20.5) |

**S1** Table [Distribution](javascript:;) of gender among first authors and senior authors on top 10 journals about liver cancer in the field of Gastroeneterology and Hepatology.
